# Economic Evaluation of ‘Watch and Wait’ Following Neoadjuvant Therapy in Locally Advanced Rectal Cancer: A Systematic Review

**DOI:** 10.1245/s10434-024-16056-4

**Published:** 2024-08-24

**Authors:** Ishraq Murshed, Zachary Bunjo, Warren Seow, Ishmam Murshed, Sergei Bedrikovetski, Michelle Thomas, Tarik Sammour

**Affiliations:** 1 Discipline of Surgery, Faculty of Health and Medical Sciences, Adelaide Medical School, University of Adelaide, Adelaide, SA Australia; 2 Colorectal Unit, Department of Surgery, Royal Adelaide Hospital, Adelaide, SA Australia

**Keywords:** Locally advanced rectal cancer, Watch & wait, Economic evaluation, Neoadjuvant therapy, Complete clinical response

## Abstract

**Background:**

Owing to multimodal treatment and complex surgery, locally advanced rectal cancer (LARC) exerts a large healthcare burden. Watch and wait (W&W) may be cost saving by removing the need for surgery and inpatient care. This systematic review seeks to identify the economic impact of W&W, compared with standard care, in patients achieving a complete clinical response (cCR) following neoadjuvant therapy for LARC.

**Methods:**

The PubMed, OVID Medline, OVID Embase, and Cochrane CENTRAL databases were systematically searched from inception to 26 April 2024. All economic evaluations (EEs) that compared W&W with standard care were included. Reporting and methodological quality was assessed using the Consolidated Health Economic Evaluation Reporting Standards (CHEERS), BMJ and Philips checklists. Narrative synthesis was performed. Primary and secondary outcomes were (incremental) cost-effectiveness ratios and the net financial cost.

**Results:**

Of 1548 studies identified, 27 were assessed for full-text eligibility and 12 studies from eight countries (2016–2024) were included. Seven cost-effectiveness analyses (complete EEs) and five cost analyses (partial EEs) utilized model-based (*n* = 7) or trial-based (*n* = 5) analytics with significant variations in methodological design and reporting quality. W&W showed consistent cost effectiveness (*n* = 7) and cost saving (*n* = 12) compared with surgery from third-party payer and patient perspectives. Critical parameters identified by uncertainty analysis were rates of local and distant recurrence in W&W, salvage surgery, perioperative mortality and utilities assigned to W&W and surgery.

**Conclusion:**

Despite heterogenous methodological design and reporting quality, W&W is likely to be cost effective and cost saving compared with standard care following cCR in LARC.

*Clinical Trials Registration* PROSPERO CRD42024513874.

**Supplementary Information:**

The online version contains supplementary material available at 10.1245/s10434-024-16056-4.

Rectal cancer exerts a large healthcare burden globally, ranking as one of the highest in prevalence and mortality worldwide.^1^ Standard management of locally advanced rectal cancer (LARC) involves neoadjuvant therapy followed by total mesorectal excision (TME).^2^ Some patients achieve a complete clinical response (cCR) post-neoadjuvant therapy, where no tumor is detectable on clinical examination, endoscopy and imaging.^3^ In such cases, TME may expose patients to perioperative morbidity, mortality, and potential long-term sexual, urinary and bowel dysfunction and be unnecessary.^4,5^ Non-operative management or ‘watch and wait’ (W&W) offers comparable disease-free survival (DFS) rates^6–11^ and, owing to avoidance of surgery, has been associated with improved quality of life (QoL) compared with TME.^12,13^ Despite the 20–25% risk of local regrowth in W&W that necessitates intensive surveillance,^10,11^ successful surgical salvage is possible in over 90% of cases.^7,11^

Total neoadjuvant therapy (TNT), combining neoadjuvant chemoradiotherapy (nCRT) and upfront chemotherapy, has demonstrated improved rates of DFS, cCR, and pathological complete response (pCR) in LARC.^14–16^ Accordingly, TNT use has increased dramatically in recent years.^17^ Given the increasing rates of cCR, risks of surgery, and similar W&W DFS, it is possible that more patients and clinicians will consider W&W as the primary treatment option.

Global treatment costs of colorectal cancer are projected to surpass billions of US dollars and a co-ordinated international effort to mitigate the rising cost is warranted.^18^ LARC management is expensive given frequent use of combination treatment modalities and complex surgery.^19^ Surgery is associated with significant costs, attributable to inpatient hospital stay, surgical supplies, operating theatre expenses, and high overall complication rates.^20^ By avoiding surgery and inpatient hospital care, W&W has the potential to be a substantially cost-effective and cost-saving intervention.^21–23^ However, to capture recurrences early, W&W protocols involve rigorous surveillance through frequent multimodal imaging, blood tests, endoscopies and office visits, which adds to the financial burden.

The objective of this systematic review was to identify the economic impact of W&W, versus standard of care, in patients who have achieved cCR following neoadjuvant therapy for LARC.

## Methods

This systematic review of healthcare economic evaluations (EEs) followed the Preferred Reporting Items for Systematic Reviews and Meta-Analyses (PRIMSA)^24^ and Synthesis without Meta-Analysis (SWiM) guidelines,^25^ and was prospectively registered with PROSPERO (CRD42024513874).

### Eligibility Criteria

Eligible participants were adult (>16 years) primary LARC patients who received neoadjuvant therapy (chemotherapy, radiotherapy, or both). The intervention studied was W&W versus standard of care (TME with or without adjuvant chemotherapy). The primary outcome was the (incremental) cost-effectiveness ratio, and secondary outcomes were the net financial costs of the interventions.

Complete (cost-effective analysis, cost-utility analysis, cost-benefit analysis, and cost-minimization analysis) and partial (cost analysis) EEs with varying healthcare perspectives, time horizons, settings, and discount rates were eligible. Exclusion criteria included pediatric population, malignancy proximal to the rectosigmoid junction, distant metastasis, or recurrent LARC. Case reports, editorials, letters, systematic reviews, comments, mini-reviews, book chapters and conference abstracts were excluded.

### Search Strategy

The PubMed, OVID Embase, OVID Medline, and Cochrane Library CENTRAL databases were searched from inception to 21 February 2024, and updated on 26 April 2024, for studies of any design, in any setting, without language or publication restrictions. Keywords were related to ‘rectal neoplasms’, ‘watchful waiting’, ‘organ preservation’, ‘economics’, and ‘cost’. The full search strategy was developed with input from a database librarian (electronic supplementary material [ESM] 1). Supplementary searching included reviewing reference lists of included articles, consulting subject experts, and screening grey literature.

### Study Selection

Studies were screened using the Covidence software (Veritas Health Innovation, Melbourne, VIC, Australia). After duplicate removal, two reviewers independently screened all titles, abstracts, and full texts, resolving disagreements by consensus, arbitrated by a third reviewer.

### Data Extraction

Baseline study characteristics and outcome data were extracted independently by two reviewers using a standardized form, resolving discrepancies by consensus, arbitrated by a third reviewer. Study characteristics included author details, publication year, country, healthcare setting, study period, type of EE, analytical approach, study perspective, time horizon, discount rate, population and intervention characteristics. Outcome data extracted were the mean costs, effectiveness, and uncertainty analysis.

### Quality Assessment

The methodological quality of trial-based EEs was examined using the British Medical Journal (BMJ) checklist,^26^ which contains 35 items evaluating study design, data collection, analysis and interpretation. Model-based EEs were assessed using the Philips checklist,^27^ which consists of 58-items assessing model structure, data, and consistency. Reporting of EEs were evaluated using the Consolidated Health Economic Evaluation Reporting Standards (CHEERS) 2022 checklist, which includes 28 items aimed to standardize and enhance transparency in reporting.^28^

Two reviewers independently assessed the methodological and reporting quality, resolving disagreements by consensus, arbitrated by a third reviewer. Each item for the CHEERS, BMJ, and Philips checklists was recorded as ‘yes’ (1 point), ‘no’ (0 points), or ‘not applicable’ to assess completeness. As there are no validated scoring metrics for these checklists, grading systems or percentage cut-offs were not used.^26–29^

### Data Analysis

Due to jurisdiction-specific factors such as location, healthcare system, time horizon, and perspective, there can be considerable heterogeneity in outcomes of EEs. Guidelines discourage pooling of primary outcomes when studies vary in their clinical setting or methodology,^30^ precluding meta-analysis due to these inherent limitations. Instead, synthesis of economic evidence followed established guidelines, employing structured narrative synthesis to present study characteristics, methodological quality, and outcomes.^25^ For review purposes, published costs were adjusted for inflation and purchasing power parity using a validated online calculator (https://eppi.ioe.ac.uk/costconversion/) using OECD data, targeting 2022 Australian dollars (AUS$) when reference data were available.^31^

### Publication *Bias*

Publication bias was assessed by searching the grey literature, conference abstracts not proceeding to publication, analysis of sponsorship in included studies and outcome differences, and presence and results of uncertainty analysis.^30^

## Results

### Study Selection

**Fig. 1:**
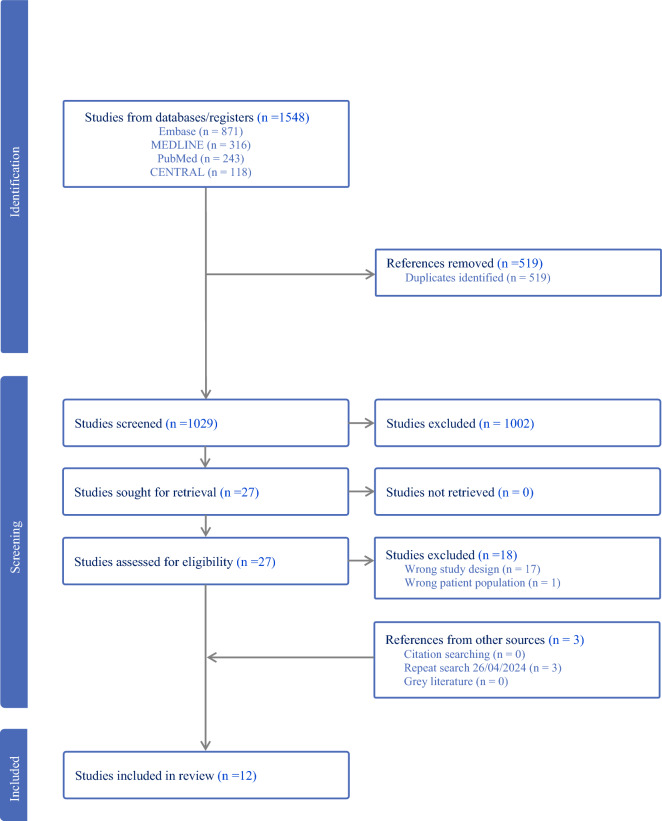
PRISMA diagram. *PRISMA* preferred reporting items for systematic reviews and meta-analyses

Of 1548 articles retrieved, 519 duplicates were removed; of the 1029 articles screened by title and abstract, 1002 were irrelevant. Twenty-seven articles proceeded to full-text review and 9 met the inclusion criteria. Studies were excluded due to wrong study design (*n* = 17) and wrong patient population (*n* = 1). Supplemental searches identified three more studies, resulting in a total of 12 studies for inclusion in this systematic review (Fig. 1).

### Economic Study Characteristics

Table 1 summarizes the characteristics of the included studies. Studies were published between 2016 and 2024 and originated from eight countries: United States,^22,23^ The Netherlands,^32,33^ Spain,^34,35^ Germany,^36,37^ Australia,^38^ New Zealand,^39^ United Kingdom,^21^ and Japan.^40^ Seven studies were cost-effectiveness analyses (CEA),^21–23,32,34,35,37^ while the remainder were cost analyses (CA).^33,36,38–40^ Seven studies adopted a model-based approach.^21–23,32,34,36,37^

**Table 1:** Characteristics of the included studies (*n* = 12)

Author (year)	Type of EE	Analytic approach	Country	Time period	Patient population/demographic	Intervention	Comparator	Funding
Cooper et al. ^38^	Cost analysis	Trial	Australia	2016–2018	Patients with rectal cancer who received neoadjuvant long-course CRT Demographic: Unspecified	W&W following cCR	Surgery (ULAR) with pCR	Not reported
Crean et al. ^39^	Cost analysis	Trial	New Zealand	2014–2021	Patients with a histological diagnosis of rectal adenocarcinoma, absence of distant metastases and treated with neoadjuvant long-course CRT Demographic: Age 64–66 years (average), mostly female (65–83%)	W&W following cCR	TME with pCR	Not reported
Cui et al.^23^	Cost-effectiveness analysis	Model	USA	RY: 2019	Patients with stage II/III rectal cancer (locally advanced T3, N any or T1-2, N1-2) with cCR after neoadjuvant CRT Demographic: Unspecified	W&W	Surgery (LAR or APR)	US Government (National Institutes of Health)
Ferri et al.^35^	Cost-effectiveness analysis	Trial	Spain	2010–2020	Locally advanced rectal cancer patients treated with neoadjuvant CRT Demographic: Age 60–70 years (average), mostly male (55–63%)	W&W following cCR	TME with pCR	Academia (Catholic University of Murcia)
Gani et al.^36^	Cost analysis	Model	Germany	Not reported	Patients with locally advanced distal rectal cancer who achieved cCR after neoadjuvant CRT Demographic: Unspecified	W&W	APR	Academia (University of Tübingen)
Hendriks^32^	Cost-effectiveness analysis	Model	Netherlands	RY: 2016	Dutch locally advanced rectal cancer at restaging following neoadjuvant CRT Demographic: Age 68 years (average), mostly male (61%)	W&W following cCR	TME following iCR	Not reported
Hupkens et al.^33^	Cost analysis	Trial	Netherlands	2004–2014	Locally advanced rectal cancer patients received neoadjuvant CRT Demographic: Age 63–66 years (average), mostly male (65%)	W&W following cCR	TME following iCR	None
Miller et al.^22^	Cost-effectiveness analysis	Model	USA	RY: 2019	Adult resectable locally advanced (T3-4N0 or node-positive) rectal adenocarcinoma who achieved complete clinical response to standard upfront neoadjuvant CRT Demographic: Age 64 years	W&W	TME (APR or LAR)	Academia (Stanford University) and Industry (Varian Medical Systems)
Rao et al.^21^	Cost-effectiveness analysis	Model	UK	RY: 2014–2015	Patients with locally advanced rectal cancer managed with neoadjuvant CRT and who achieved cCR Demographic: 3 cohorts—60-year-old male, no comorbidities; 80-year-old male, mild comorbidities (Charlson score <3); 80-year-old male, significant comorbidities (Charlson score >3)	W&W	Radical surgery	UK Government (National Institutes of Health)
Rodriquez-Pascual et al.^34^	Cost-effectiveness analysis	Model	Spain	RY: 2019	Patients with locally advanced (stage II and III) rectal cancer received neoadjuvant CRT and achieved cCR Demographic: Unspecified	W&W	Standard rectal resection, robotic rectal resection	Not reported
Sawada et al.^40^	Cost analysis	Trial	Japan	2014–2021	Patients with stage II–III low rectal cancer treated with neoadjuvant CRT Demographic: Majority age >65 years, and male	W&W following cCR	APR following iCR	Not reported
Wurschi et al.^37^	Cos- effectiveness analysis	Model	Germany	RY: 2021	Patients with stage III rectal cancer (T3/4, N+, CRM+) in the lower third of the rectum (<6 cm from the anal verge) received neoadjuvant treatment Demographic: Majority age >65 years (65%), and male (62%)	TNT, cCR then W&W	CRT then APR	None

*EE* economic evaluation, *RY* reference year, *TME* total mesorectal excision, *APR* abdominoperineal resection, *LAR* low anterior resection, *ULAR* ultra-low anterior resection, *CRT* chemoradiotherapy, *cCR* complete clinical response, *pCR* complete pathological response, *iCR* incomplete clinical response, *W&W* watch and wait protocol, *TNT* total neoadjuvant therapy

Comparators to W&W were abdominoperineal resection (APR) in three studies,^36,37,40^ APR and low anterior resection (LAR) in three studies,^22,23,38^ and unspecified in the remaining studies. Rodriguez-Pascual et al. compared W&W with standard and robotic resection.^34^ In the article by Wurschi et al., the W&W cohort received TNT whereas the surgical comparator received nCRT.^37^ For the remainder, both cohorts received nCRT. Three studies specified low rectal cancer requiring APR,^36,37,40^ while the remaining studies investigated rectal cancer of any height. In all studies, only patients with cCR were offered W&W, whereas patients undergoing surgery may have achieved pCR,^35,38,39^ incomplete clinical response,^32,33,37,40^ or cCR,^21–23,34,36^ potentially biasing the outcomes.

### Methodological Details

Table 2 summarizes the methodological details of the included studies. Eleven studies adopted a hospital or third-party payer (TPP) perspective,^21–23,32–36,38–40^ while Wurschi et al. adopted a patient perspective.^37^ When unspecified, perspective was inferred from costing information. Various time horizons were explored: 2 years,^33^ 3 years,^35,38^ 5 years,^23,36,37,39,40^ and lifetime.^21,22,32,34^ Costs and effects were discounted in seven studies, ranging from 1.5 to 4% depending on study jurisdiction.^21–23,32,34,36,40^ Model types included decision tree,^36^ Markov,^22,23,32,34,37^ or both.^21^

**Table 2:** Methodological quality and details of the included economic evaluations (*n* = 12)

Author (year)	Type of EE	Perspective	Time horizon (years)	Discount rate for costs (%)	Discount rate for effects (%)	Inflation adjustment (Y/N)	Data source of resource use	Data source of cost estimate	Data source of effects	Outcome measure(s)	Uncertainty analysis	CHEERS score (%)	BMJ/Philips score (%)
Cooper et al.^38^	CA	TPP^a^	3	–	NA	N	Medical records, local institution policy	Hospital database (North Metropolitan Health Service), 2020 Australian MBS and PBS	NA	Total cost per patient	None	57	68
Crean et al.^39^	CA	TPP^a^	5^a^	–	NA	N	Electronic medical records, local institution policy	Hospital database (District Health Board’s Planning and Finance Department)	NA	Total cost per patient	*p*-Value	48	64
Cui et al.^23^	CEA	TPP	5	3	3	Y	Published literature, US Social Security life tables	2019 US Physician’s Fee Schedule	Published literature	Incremental cost, incremental QALY, ICER	Deterministic (one-way) and probabilistic SA	82	83
Ferri et al.^35^	CEA	TPP^a^	3^a^	–	–	N	Medical records, local institution policy	Institute of Validation of Efficacy Clinic (IVEC) of the HM Hospitals group	Institutional database	Incremental cost, incremental QALY, ICER	Subgroup analysis, 95% CI, standard deviation, *p*-values	69	64
Gani et al.^36^	CA	TPP	5	3.5	NA	N	Clinical trial protocol	“Gebührenordnung für Ärzte” (GOÄ) German billing system	NA	Total cost per patient	Deterministic (one-way) SA	80	60
Hendriks^32^	CEA	TPP^a^	Lifetime	4	1.5	N	Statistics Netherlands, published literature, Dutch clinical practice guidelines	Average tariffs of three health insurance companies with all Dutch hospitals	Expert elicitation, published literature	Incremental cost, incremental QALY, ICER	Scenario analysis, deterministic (one-way) SA	75	72
Hupkens et al.^33^	CA	Hospital	2	–	NA	N	Medical records, clinical trial protocol	Reference 2014 Dutch guideline prices, hospital database	NA	Total cost per patient	Standard deviations	70	82
Miller et al.^22^	CEA	TPP	Lifetime	3	3	Y	Published literature, surveillance schedule of clinical trial	US Medicare inpatient and home healthcare prospective payment systems, published literature, US 2019 Medicare fee schedule	Published literature	Incremental cost, incremental QALY, ICER	Deterministic (one-way) and probabilistic SA	79	81
Rao et al.^21^	CEA	TPP	Lifetime	3.5	3.5	N	Published literature, surveillance schedule of clinical trial	UK NHS reference costs 2014–2015	Published literature	Incremental cost, incremental QALY	Deterministic and probabilistic SA, subgroup analysis, scenario analysis, 95% CI, standard deviation	86	87
Rodriquez-Pascual et al.^34^	CEA	TPP	Lifetime	3	3	N	Institutional database, published literature	Published literature	Published literature, institutional database	Incremental cost, incremental QALY, ICER	Probabilistic SA	71	61
Sawada et al.^40^	CA	TPP^a^	5	–	NA	N	Medical records	Medical Fee Points of Japan 2020	NA	Total cost per patient	None	48	55
Wurschi et al.^37^	CEA	Patient	5	3.5	3.5	N	Published literature, surveillance schedule of clinical trial	German statutory health insurance	Published literature	Incremental cost, incremental QALY, ICER	Deterministic SA, subgroup analysis	86	69

^a^Unspecified in the study, derived from costing information provided

*EE* economic evaluation, *CA* cost analysis, *CEA* cost-effectiveness analysis, *TPP* third-party payer, *NA* not applicable, *QALY* quality-adjusted life-year, *ICER* incremental cost-effectiveness ratio, *SA* sensitivity analysis, *MBS* Medicare Benefits Schedule, *PBS* Pharmaceutical Benefits Scheme, *NHS* National Health Service, *Y* yes, *N* no, *CHEERS* Consolidated Health Economic Evaluation Reporting Standards, *CI* confidence interval

Resource use in trial-based EEs was estimated from patient records and local institutional policies. Transition probabilities in model-based EEs were sourced from published literature, population statistics, practice guidelines, clinical trials, and institutional databases. Cost estimates reflected jurisdiction-specific payment systems, except in one Spanish study, where costs were derived from the US.^34^ Utilities were predominantly sourced from published literature and also institutional databases in two studies^34,35^ and expert elicitation in one study.^32^ Detailed data sources for modeling studies are provided in Table 3.

**Table 3:** Input parameters for modeling studies (*n* = 7)

Author (year)	Model type	Data source of transition probabilities (study type)	Data source of health utility states (study type)	Data source of costing estimates (study type)
Cui et al.^23^	Markov microsimulation	Dossa ^11^ (MA), van der Valk ^7^ (pros cohort], Smith ^46^ (retro cohort), Rao ^21^ (model), Marijnen ^47^ (RCT), Miller ^22^ (model), Ikoma ^48^ (retro cohort)	Couwenberg ^49^ (cross-sectional, rectal cancer), van den Brink ^50^ (cross-sectional, rectal cancer)	Physician’s Fee Schedule,^51^ Raldow ^52^ (model), DMEPOS Schedule ^53^, Miller ^22^ (model), Duncan ^54^ (CA)
Gani et al.^36^	Decision tree	Martens ^55^ (cohort), Maas ^8^ (cohort), Renehan ^9^ (propensity cohort), Appelt ^56^ (cohort), Maas ^57^ (pooled cohort IPD analysis)	NA	“Gebührenordnung für Ärzte” (GOÄ)^58^
Hendriks ^32^	Markov microsimulation	Maas ^57^ (pooled cohort IPD), Maas ^59^ (pros cohort), Beets ^60^ (cohort), Habr-Gama ^61^ (cohort), Marijnen ^47^ (RCT), van den Brink ^50^ (RCT), Nielsen ^62^ (SR)	Expert elicitation, Statistics Netherlands ^32^, Ness ^63^ (cross-sectional, interview utility)	CZ, Menzis, Avero Achmea Health Insurance companies ^32^
Miller et al.^22^	Markov microsimulation	Li ^64^ (SR), Smith ^46^ (retro cohort), van den Valk ^7^ (pros cohort), Maas ^8^ (cohort), Renehan ^9^ (propensity cohort), Habr-Gama ^61^ (cohort), Valentini ^65^ (pros cohort), Guren ^66^ (SR), Ikoma ^48^ (retro cohort), Moghadamyeghaneh ^67^ (national cohort), Mohiuddin ^68^ (cohort), Global Health Observatory ^69^, Goldberg ^70^ (RCT)	Hupkens ^13^ (matched cohort study), van den Brink ^50^ (cross-sectional, rectal cancer)	Medicare Inpatient and Home Healthcare Prospective Payment Systems, Berger ^71^ (RCT), Medicare Fee Schedule^51^
Rao et al.^21^	Decision tree model, Markov microsimulation	Habr-Gama ^6^ (pros cohort), Smith ^72^ (model), Smith ^73^ (SR), Neuman ^74^ (model), Martin ^75^ (SR), Valentini ^76^ (model), Maas ^57^ (pooled cohort IPD analysis), Guillem ^77^ (pros cohort), Garcia-Aguilar ^78^ (pros cohort), Tepper ^79^ (RCT), van den Brink^80^ (RCT), NHS Statistics ^81^, Hahnloser ^82^ (retro cohort), Cassidy ^83^ (RCT)	Neuman ^74^ (model), Konski ^84^ (model, prostate cancer), van den Brink ^50^ (cross-sectional, rectal cancer), Miller ^22^ (model)	NHS reference costs 2014–2015^85^
Rodriquez-Pascual et al.^34^	Markov microsimulation	Li ^64^ (SR), Smith ^46^ (retro cohort), van der Valk ^7^ (pros cohort), Habr-Gama ^61^ (cohort), Renehan ^9^ (propensity cohort)	van der Brink ^50^ (cross-sectional, rectal cancer), Quijano ^86^ (pros cohort)	Miller ^22^ (model)
Wurschi et al.^37^	Markov microsimulation	Garcia-Aguilar ^87^ (RCT), Marijnen ^47^ (RCT), van der Valk^7^ (pros cohort), Verheij ^88^ (RCT), Ikoma ^48^ (retro cohort), Dossa ^11^ (MA), Rao ^21^ (model), Rodel ^89^ (RCT), Diefenhardt ^90^ (retro cohort), German population statistics^91^	Couwenberg ^49^ (cross-sectional, rectal cancer), van den Brink ^50^ (cross-sectional, rectal CA), Cui ^23^ (model), Kosmala ^92^ (RCT)	German-published statistics on income, employment, co-payments and distance traveled^91^

*MA* meta-analysis, *pros cohort* prospective cohort study, *retro cohort* retrospective cohort study, *model* decision modeling study, *RCT* randomized controlled trial, *IPD* individual patient data, *SR* systematic review, *CA* cost analysis, *NHS* National Health Service, *NA* not applicable

Model-based uncertainty was assessed with deterministic sensitivity analysis (DSA) to investigate parameter uncertainty in six studies,^21–23,32,36,37^ probabilistic sensitivity analysis (PSA) to investigate simultaneous joint parameter uncertainty in four studies,^21–23,34^ and scenario analysis to investigate model assumptions in three studies.^21,23,32^ Subgroup analyses were conducted in three studies to investigate heterogeneity.^21,35,37^ Three trial-based EEs reported statistical analysis: standard deviations,^33^ *p*-values,^39^ or both.^35^

### Quality Assessment

**Fig. 2:**
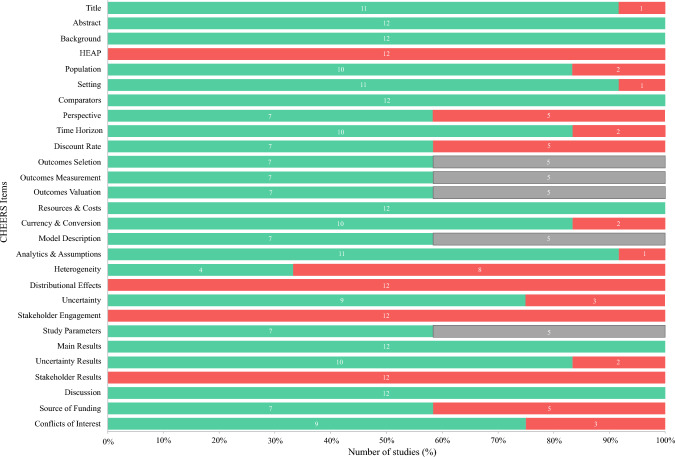
Number of studies reporting CHEERS items (green, yes; red, no; grey; NA). *CHEERS* consolidated health economic evaluation reporting standards consolidated health economic evaluation reporting standards, *NA* not available

Studies were heterogenous in reporting and methodological quality. Table 2 displays the assessment for each study. The completeness of reporting ranged between 48 and 86% using the CHEERS 2022 checklist. Figure 2 demonstrates the proportion of studies that satisfied each item in the checklist. Completeness for the BMJ checklist for trial-based EEs was between 55 and 82%, and between 60 and 87% for the Philips checklist for model-based EEs. The full quality assessment matrix for each study is displayed in ESM 2.

### Cost Effectiveness

Seven studies (six modeling, one trial) evaluated cost effectiveness.^21–23,32,34,35,37^ Outcomes are summarized in Table 4 for model-based EEs and in Table 5 for trial-based EEs. Across time horizons of 3 years to lifetime, all studies consistently identified W&W to be dominant over surgical comparators, offering lower costs and higher quality-adjusted life-years (QALYs) from both TPP^21–23,32,34,35^ and patient perspectives.^37^ Standardized incremental costs of W&W ranged from AUS$1141 to AUS$192,145 (3–50%) less per patient (i.e. cost saving) from the TPP perspective and AUS$3203 (25%) less from the patient perspective. The incremental QALYs associated with W&W ranged from 0.089 to 2.03 more QALYs than surgery.

**Table 4:** Summary of primary outcomes—model-based (*n* = 7)

Author (year)	Perspective	Time horizon (years)	Country, currency (reference year)	Base-case cost: W&W and surgery	Base-case QALY: W&W and surgery	Incremental cost	Incremental QALY	Result	Adjusted cost (AUS$, 2022)	Adjusted cost difference in AUS$, 2022 (% difference)	Uncertainty analysis results
*Complete economic evaluations (n = 6)*											
Cui et al.^23^	TPP	5	USA, US$ (2019)	W&W: 26,500 LAR: 50,485	W&W: 3.41 LAR: 3.24	−23,985	+0.170	ICER: W&W dominant vs. APR and LAR	W&W: 41,154 LAR: 78,402	−37,248 (48% cheaper)	*DSA:* Model sensitive to 2-year local regrowth and 5-year distant recurrence rate following W&W, LAR (post-ostomy reversal), APR utilities *PSA:* W&W dominant in 100% of simulations *Scenario:* Adjuvant chemotherapy timings did not affect outcome
				W&W: 23,895 APR: 40,655	W&W: 3.40 APR: 3.17	−16,761	+0.230		W&W: 37,108 APR: 63,136	−26,028 (41% cheaper)	
Hendriks^32^	TPP^a^	Lifetime	Netherlands, € (2016)	W&W: 16,537 TME: 17,048	W&W: 6.391 TME: 6.302	−511	+0.089	ICER: W&W dominant	W&W: 36,929 TME: 38,070	−1141 (3% cheaper)	*DSA:* Model sensitivity to W&W and post-TME utilities *Scenario:* W&W dominant when compared with scenarios from two clinical trials (Maas, Habr-Gama)
Miller et al.^22^	TPP	Lifetime	USA,US$ (2019)	W&W: 31,800 LAR: 60,200	W&W: 12.652 LAR: 12.125	−28,400	+0.527	ICER: W&W dominant over LAR and APR	W&W: 49,384 LAR: 93,489	−44,105 (47% cheaper)	*DSA:* Model sensitive to rates of local recurrence and distant metastasis in W&W, salvage rates of W&W, disutility of TME *PSA:* W&W dominant over LAR in 99.5% and APR in 99.8% of simulations
				W&W: 31,800 APR: 63,800	W&W: 12.652 APR: 12.050	−32,000	+0.601		W&W: 49,384 APR: 99,080	−49,696 (50% cheaper)	
Rao et al.^21^	TPP	Lifetime	UK, £ (2014)	NR. Incremental cost and QALY for a healthy 60-year-old male		−8095	+0.63	ICER: W&W dominant over radical surgery in all patient populations examined	N/A	−20,651	*DSA:* Model sensitive to rates of recurrence following W&W or surgery, utilities of W&W and surgery and perioperative mortality *PSA:* W&W cost effective with high certainty (>70%). More cost effective with increasing age and comorbidities of the cohort *Scenario:* W&W cheaper in all cohorts with high certainty (70–85%) when modeled using NCCN guidelines
				NR. Incremental cost and QALY for a healthy 80-year-old male:		−6274	+0.56		NA	−16,005	
				NR. Incremental cost & QALY for comorbid 80-year-old male		−7290	+0.72		NA	−18,597	
Rodriquez-Pascual et al.^34^	TPP	Lifetime	Spain, € (2019)	NR. Incremental cost and QALY for W&W vs. standard rectal resection:		−74,857	+2.03	ICER: W&W dominant over standard and robotic resection	NA	−191,720	*PSA:* W&W dominant in costs in 99.98% of simulations. W&W dominant in QALY over standard resection in 86.9% and robotic resection in 55% of simulations
				NR. Incremental cost and QALY for W&W vs. robotic rectal resection.		−75,023	+0.44		N/A	−192,145	
Wurschi et al.^37^	Patient	5	Germany, € (2021)	W&W: 4711 APR: 6252	W&W: 3.87 APR: 3.23	−1540	+0.640	ICER: W&W dominant over APR	W&W: 9790 APR: 12,993	−3203 (25% cheaper)	*DSA:* Model sensitive to patient cost of W&W and utility difference between W&W and APR *Subgroup:* For retired patients, model only sensitive to utility difference
*Partial economic evaluations (n = 1)*											
Gani et al.^36^	TPP	5	Germany, € (NR)	W&W: 6344 APR: 14,511	NA	−8167	NA	W&W cheaper than APR	NA	NA	*DSA:* W&W cost saving unless MRI + endoscopy >€955 per visit

^a^Unspecified in the study, derived from costing information provided

*TPP* third-party payer, *W&W* Watch and Wait, *NR* not reported, *NA* not applicable, *QALY* quality-adjusted life-year, *ICER* incremental cost-effectiveness ratio, *APR* abdominoperineal resection, *LAR* low anterior resection, *DSA* deterministic sensitivity analysis, *PSA* probabilistic sensitivity analysis, *NCCN* National Comprehensive Cancer Network, *MRI* magnetic resonance imaging, *TME* total mesorectal excision, *€* Euros, *£* Great Britain pound, *US$* United States dollar

**Table 5:** Summary of primary outcomes—trial-based (*n* = 5)

Author (year)	Perspective	Time horizon (years)	Country, currency (reference year)	Mean cost: W&W and surgery (*n*)	Mean QALY: W&W and surgery (*n*)	Incremental cost	Incremental QALY	Result	Adjusted cost (AUS$, 2022)	Adjusted cost difference in AUS$, 2022 (% difference)	Uncertainty analysis
*Complete economic evaluations (n = 1)*											
Ferri et al.^35^	TPP^a^	3	Spain, € (NR)	W&W: 30,008 (*n* = 40) Surgery: 34,447 (*n* = 40)	W&W: 2.44 (*n* = 40) Surgery: 2.18 (*n* = 40)	−4 439	+0.260	ICER: W&W dominant	NA	NA	95% CI for costs: W&W always less costly 95% CI for utility: 7.11% crossover of W&W and surgery. *p*-Value 0.001 and 0.017 for difference in cost and QALY
*Partial economic evaluations (n = 4)*											
Cooper et al. ^38^	TPP^a^	3	Australia, AUS$ (2020)	W&W: 48,549 (*n* = 5) Surgery: 81,032 (*n* = 5)	N/A	−32,483	N/A	W&W cheaper	W&W: 55,315 Surgery: 92,325	−37,010 (40% cheaper)	None
Crean et al.^39^	TPP^a^	5	New Zealand, NZ$ (NR)	W&W: 47,906 (*n* = 23) Surgery: 70,760 (*n* = 38)	NA	−22,854	NA	W&W cheaper	NA	NA	*p*-Value of cost difference between W&W and surgical group was 0.014
Hupkens et al.^33^	Hospital	2	Netherlands, € (2014)	W&W: 6713 (*n* = 105) Surgery: 17,108 (*n* = 187)	NA	−10,395	NA	W&W cheaper	Surgery: 38,675	−23,499 (61% cheaper)	Standard deviation of costs for W&W was €2964, and for surgery was €10,450
Sawada et al.^40^	TPP^a^	5	Japan, ¥ (2020)	W&W: 1,305,450 (*n* = 15) Surgery: 2547 760 (*n* = 36)	NA	−1,242,310	NA	W&W cheaper	Surgery: 36,803	−17,945 (49% cheaper)	None

^a^Unspecified in the study, derived from costing information provided

*TPP* third-party payer, *W&W* Watch and Wait, *NR* not reported, *NA* not applicable, *QALY* quality-adjusted life-year, *ICER* incremental cost-effectiveness ratio, *CI* confidence interval, *€* Euros, *AUS$* Australian dollars, *¥* Japanese yen

In US studies over 5-year and lifetime horizons, W&W demonstrated 40–50% lower costs and superior QALY compared with both LAR and APR.^22,23^ Similarly, Spanish studies over 3-year and lifetime horizons demonstrated W&W dominance over surgical resection, including robotic resection in one study.^34,35^ Ferri et al. raised methodological concern by appearing to apply utilities derived from Short-Form 36 (SF-36) questionnaires at 12 months across 3 years without elaboration, potentially affecting the reliability of QALY estimates.^35^ Rodriguez-Pascual et al. used US costing estimates, potentially limiting applicability to the Spanish jurisdiction.^34^ Dutch and UK studies found W&W to be dominant over surgery over a lifetime,^21,32^ despite challenges in obtaining appropriate W&W utilities due to the lack of published literature. Hendriks relied on expert elicitation at the author’s institution^32^ and Rao et al. relied on proxy data from prostate cancer literature.^21^

One German study provided the only patient perspective, demonstrating W&W dominance over APR across a 5-year time horizon.^37^ However, W&W patients received TNT versus nCRT in the APR comparator, potentially limiting applicability as this may not reflect clinical practice.

### Cost

Five studies performed CA comparing W&W with surgery; four trial-based EEs^33,38–40^ and one model-based EE.^36^ All were performed from TPP or hospital perspectives, with time horizons ranging from 2 to 5 years. W&W consistently showed lower costs compared with surgery across all studies. Standardized cost differences ranged from AUS$17,945 to AUS$37,010 (40–61% less costly).

A Dutch study with a 2-year time horizon demonstrated mean hospital costs of AUS$15,176 (95% confidence interval [CI] $13,895–16,456) for W&W and AUS$38,675 (95% CI $35,291–42,060) for surgery, translating to an AUS$23,499 (61%) cost reduction per patient.^33^ Similarly, a small (*n* = 10) trial-based Australian study with a 3-year time horizon showed mean costs of AUS$55,315 for W&W and AUS$92,325 for surgery, resulting in an AUS$37,010 (40%) cost reduction per patient.^38^

Three studies compared costs with a 5-year time horizon. In Japan, mean costs were AUS$18,858 for W&W and AUS$36,803 for APR, with a cost saving of AUS$17,945 (49%) per patient.^40^ In New Zealand, mean costs were NZ$47,906 for W&W and NZ$70,760 for surgery, resulting in a NZ$22,854 (32%) cost saving per patient.^39^ This was the only study to factor in neoadjuvant treatment costs, potentially increasing net financial costs compared with other studies. A German decision tree model found W&W costs were €6344 and APR costs were €14,511, saving €8167 (56%) per patient.^36^

The varying approaches used with respect to time horizon, discounting, and management of inflation limited the value of across-study comparisons of absolute and incremental costs.

### Heterogeneity

Three studies performed subgroup analyses. Ferri et al. found greater cost savings with the W&W approach for low rectal tumors compared with medium-high rectal tumors, likely due to increased surgical complexity and risk of post-surgical complications.^35^ Wurschi et al. examined patient costs and found employed W&W patients had twice the cost saving compared with retired patients, likely due to reduced productivity losses.^37^ Rao et al. examined three cohorts: healthy 60- and 80-year-old males, and comorbid 80-year-old males, finding W&W dominant across all cohorts.^21^

### Uncertainty

Ten studies conducted statistical or sensitivity analysis to address uncertainty.^21–23,32–37,39^ DSA across six studies demonstrated key parameters impacting outcomes were rates of local regrowth^21–23^ and distant metastasis following W&W,^22,23^ salvage surgery,^22^ perioperative mortality^21^ and utilities for W&W and surgical comparators.^21–23,32,37^ Doubling W&W costs or decreasing surgical costs by 90% could have altered outcomes in two studies.^36,37^

PSA was performed in four studies.^21–23,34^ US studies demonstrated W&W to be dominant over 5-year and lifetime horizons in almost all simulations.^22,23^ Rao et al. demonstrated W&W’s dominance with high certainty (>70%), with increasing certainty among older and comorbid patients.^21^ Rodriguez-Pascual et al. showed W&W to be cost saving in almost all simulations and mostly increasing QALYs when compared with standard and robotic resection (87% and 55% of simulations, respectively), however there were concerns regarding costing data and utility acquisition methods.^34^

Three model-based studies performed scenario analyses, examining different patient populations,^32^ adjuvant chemotherapy timings,^23^ and surveillance protocols,^21^ all showing W&W dominance in all scenarios examined. Trial-based EEs demonstrated statistically significant results, with *p*-values <0.05 in two studies^35,39^ and non-overlap of calculated confidence intervals in one study.^33^

### Publication *Bias*

Searching of the grey literature and conference abstracts not proceeding to publication did not reveal any undiscovered articles. Analysis of sponsorship demonstrated three articles with academic affiliations,^22,35,36^ two with government affiliations,^21,23^ and one with industry affiliation.^22^ There were no differences in outcome based on sponsorship, and all sponsored articles included appropriate uncertainty analysis.

## Discussion

EE is crucial in the assessment of new health technologies and health protocol implementation, enabling decision makers and policy developers to review the impact of interventions and allocate scarce healthcare resources efficiently. In this first systematic review of the global economic impact of W&W, 12 eligible studies of varying reporting quality and methodological designs were identified. Seven CEAs and five CAs all reported improved cost-effectiveness and cost-saving associated with W&W across a variety of time horizons and perspectives.

A key strength of this review was the comprehensive search strategy and broad eligibility criteria facilitating inclusion of different types of EEs globally. A consistent direction of effect favoring W&W as the dominant strategy suggests that the conclusions could be applicable internationally across heterogeneous health systems and patient populations. Multidimensional assessment of methodological and reporting quality allowed recognition and highlighting of high-quality EEs.

Of the 12 studies, 11 reported costs from TPP or hospital perspectives, while only one utilized the patient perspective. None comprehensively assessed societal costs, including indirect and intangible costs, such as productivity loss associated with frequent appointments during intensive W&W surveillance, or the psychological burden associated with the uncertainty of cancer prognosis. Additionally, none assessed implementation and maintenance costs of W&W pathways that may require significant coordination of the patient’s clinical journey, frequently necessitating a dedicated cancer care coordinator.^41^ Further research should explore the impact of a broader perspective and of broader cost considerations for W&W on its cost effectiveness. However, as two studies indicated that large changes in costs would be required to alter the conclusion of cost effectiveness of W&W versus surgery, even accounting for a more costly W&W pathway may not alter the dominance of W&W.

Considering the applicability of the results of this review to current LARC management is important. Neoadjuvant therapy included TNT in only one study.^37^ Given current practice recommendations and trends towards adoption of TNT,^2,17^ with more intensive upfront chemotherapy use and higher cCR rates, it remains unclear if the cost effectiveness of W&W will persist in TNT patient populations. In the single study utilizing TNT prior to W&W, the surgical comparator received nCRT, limiting its relevance to real-world practice, where clinicians are deciding on W&W for patients treated with TNT alone.^37^ Model-based CEAs examining TNT versus nCRT followed by TME from a TPP perspective found TNT was the dominant strategy over a 5-year time horizon,^42,43^ but did not explore the impact of W&W policies in the cohort. Finally, trial-based EE comparators in this review varied, and given incomplete clinical response is associated with poorer oncological outcomes compared with cCR, comparisons of cCR in W&W to incomplete clinical response in surgery may have biased results towards W&W. Additionally, comparison of pCR in surgery with cCR in W&W may have biased results towards surgery. Given it may be impractical or impossible to randomize patients to W&W versus surgery, high-quality prospective cohort data with standardized comparators are needed to allow accurate decision making.

This systematic review has limitations. First, the jurisdiction-specific nature of EEs results in inherent heterogeneity. This trade-off between local applicability and global generalizability precludes formal meta-analysis. As such, results were narratively synthesized, with a consistent direction of effect favoring W&W across all evaluations. Methodological and reporting quality varied significantly, with several studies failing to follow the majority of reporting guidelines.^28^ Future research should prioritize adherence to minimum reporting standards and good practice guidelines to improve quality, standardization, and transparency.

A limitation of summarizing outcomes of model-based EEs is that many used the same sources, or other model-based EEs, for their input parameters—namely transition probabilities and health state utilities. Given analogous inputs, it may not be surprising that the results themselves tended towards similar outcomes. Therefore, if an evidence source that underpins multiple EEs is inaccurate, there is concern that the multiplicity of analyses may amplify an erroneous conclusion rather than provide independent verification, reinforcing the need for thorough critical appraisal of the underlying input sources in addition to the economic modeling methodology. Moreover, due to limited literature, some studies used health utility data derived from prostate cancer literature or expert opinions, potentially introducing bias. Future research focusing on patient-reported outcomes and QoL would improve the accuracy of these decision-making models. Nevertheless, the identification of model structure and relevant input parameters may inform future model development.

In half of the model-based CEAs, DSA suggested potential outcome differences with varying local and distant recurrence rates post W&W. Although the thresholds exceeded published rates, long-term prospective follow-up data are limited. Additionally, recent literature suggests local recurrence following cCR may be a significant and independent risk factor for distant metastasis and that leaving the undetectable primary tumor in situ until recurrence occurs may result in poorer oncological outcomes.^44,45^ Despite salvage surgery being successful in almost all cases of local regrowth, more extensive surgeries may be required to achieve adequate local control. All DSAs suggested patient utilities for W&W and post-surgery may have changed the model outcome, highlighting the need for high-quality studies to refine these key parameters. Despite these limitations, PSA consistently supported W&W as the dominant strategy with high certainty.

Quality assessment tools in EE have several inherent pitfalls. No standardized tool exists, leading to the development of multiple checklists.^29^ The BMJ and Philips checklists were chosen as they were the most commonly used for trial- and model-based EEs, respectively;^29^ however, the subjective nature of these checklists results in high interrater variability, limiting the ability to provide reliable and consistent results.^29^ In this review, two independent reviewers performed each element of quality assessment, and disagreements were resolved either by consensus or a third reviewer, which helped reduce bias and systematic errors.^30^ Because no validated scoring systems of these checklists exist, it is important to emphasize that scores do not imply quality.^26–29^ Therefore, grading systems or arbitrary percentage cut-offs were not employed; instead, ESM 2 presents the complete matrix of quality assessment.

The results of this systematic review on the economic impact of W&W following neoadjuvant therapy for LARC suggest that W&W is likely cost effective and cost saving compared with surgery; however, caution is warranted given the small number of studies, clinical heterogeneity, and variable methodological quality of the included studies. Given these considerations, shared patient/clinician decision making is imperative. Nevertheless, our findings may aid the development of new decision-making models and in healthcare resource planning. Future research on patient-relevant health outcomes and societal cost effectiveness of W&W, particularly in the setting of TNT, are needed to further inform patients, clinicians, and policy makers.

## Supplementary Information

Below is the link to the electronic supplementary material.

Supplementary file1 (DOCX 44 KB)

## Data Availability

All template data collection forms, data extracted from included studies, data used for analysis, and other material used in the review may be obtained from the corresponding author upon reasonable request.
