# Supplementary material for: Economic Evaluation of ‘Watch and Wait’ Following Neoadjuvant Therapy in Locally Advanced Rectal Cancer: A Systematic Review
Source: Ann Surg Oncol. 2024 Aug 24;32(1):137–57. doi: 10.1245/s10434-024-16056-4 (PMC11659367; doi:10.1245/s10434-024-16056-4)
Supplement: Supplementary file 1 — Supplementary file1 (DOCX 44 KB) [file 10434_2024_16056_MOESM1_ESM.docx]

**Supplement 1 – Search terms**

**PubMed (Inception – 26/04/2024): 243**

("rectal neoplasms"[mh] OR rectal neoplas*[tiab] OR rectum neoplas*[tiab] OR rectal cancer*[tiab] or rectum cancer*[tiab] OR rectal tumo*[tiab] OR rectum tumo*[tiab] OR rectal malignan*[tiab] OR rectum malignan*[tiab] OR rectal adenoca*[tiab] OR rectum adenoca*[tiab] OR “cancer rectum”[tiab:~2])

AND
(“watchful waiting”[mh] OR “organ preservation”[mh] OR “conservative treatment”[mh] OR “organ sparing treatments”[mh] OR watchful waiting[tiab] OR conserve*[tiab] OR “watch wait”[tiab:~1] OR “watch see”[tiab:~1] OR non operat*[tiab] OR non surg*[tiab] OR nonoperat*[tiab] OR nonsurg*[tiab] OR organ preserv*[tiab] OR organ spar*[tiab] OR “deferral surgery”[tiab:~1] OR active surveillance[tiab] OR complete clinical[tiab] OR expectant*[tiab])

AND
(“Economics”[mh] OR economic*[tiab] OR financ*[tiab] OR cost*[tiab] OR commerc*[tiab] OR price*[tiab] OR fiscal*[tiab] OR charg*[tiab] OR pay[tiab] OR valu*[tiab] OR worth*[tiab])

**Embase OVID (Inception – 26/04/2024): 871**

Exp rectum tumour OR ((rectal OR rectum) ADJ (neoplas* OR cancer* OR tumo?r OR malignan* OR adenoca* OR carcinoma*)).ti,ab OR (cancer ADJ3 rectum).ti,ab

AND

Exp conservative treatment OR Exp organ preservation OR (conservative* OR non operat* OR non surg* OR nonoperat* OR nonsurg* OR (Organ ADJ (sparing OR preserv*)) OR watchful waiting OR (watch ADJ2 (wait OR see)) OR expectant* OR (deferral ADJ2 surgery) OR active surveillance OR complete clinical* OR expectant*).ti,ab

AND

Exp economic parameters OR Exp economic model OR (Economic* OR financ* OR cost* OR commerc* OR price* OR fiscal* OR charg* OR pay* OR valu* OR worth* OR expense* OR afford*).ti,ab

**Medline OVID (Inception – 26/04/2024): 316**

exp Rectal Neoplasms OR ((rectal OR rectum) ADJ (neoplas* OR cancer* OR tumo?r OR malignan* OR adenoca* OR carcinoma*)).ti,ab OR (cancer ADJ3 rectum).ti,ab

AND

exp Watchful Waiting OR exp Conservative Treatment OR exp Organ Preservation OR exp Organ Sparing Treatments OR (watchful waiting OR conservative* OR (organ ADJ (preserv* OR sparing)) OR non operat* OR non surg* OR nonoperat* OR nonsurg* OR (watch ADJ2 (wait OR see)) OR active surveillance OR complete clinical* OR expectant*).ti,ab

AND

Exp Economics OR (economic* OR financ* OR cost* OR commerc* OR price* OR fiscal* OR charg* OR pay* OR valu* OR worth* OR expense* OR afford*).ti,ab

**Cochrane Library CENTRAL (Inception – 26/04/2024): 118**

[mh “Rectal Neoplasms”] OR ((rectal OR rectum) NEXT (neoplas* OR cancer* OR tumo?r OR malignan* OR adenoca* OR carcinoma*)):ti,ab OR (cancer NEAR/3 rectum):ti,ab

AND

[mh “Watchful Waiting”] OR [mh “Conservative Treatment”] OR [mh “Organ Preservation”] OR [mh “Organ Sparing Treatments”] OR (“watchful waiting” OR conservative* OR (organ NEXT (preserv* OR sparing)) OR non operat* OR non surg* OR nonoperat* OR nonsurg* OR (watch NEAR/3 (wait OR see)) OR “active surveillance” OR (complete NEXT clinical*) OR expectant*):ti,ab

AND

[mh Economics] OR (economic* OR financ* OR cost* OR commerc* OR price* OR fiscal* OR charg* OR pay* OR valu* OR worth* OR expense* OR afford*):ti,ab

**Supplement 2 – Full Quality Assessment**

| **CHEERS Item** | | **Cooper 2022** | **Crean 2024** | **Cui 2022** | **Ferri 2023** | **Gani 2018** | **Hendriks 2016** | **Hupkens 2020** | **Miller 2020** | **Rao 2017** | **Rodriguez-Pascual 2022** | **Sawada 2023** | **Wurschi 2024** |
| --- | --- | --- | --- | --- | --- | --- | --- | --- | --- | --- | --- | --- | --- |
|  |  |  |  |  |  |  |  |  |  |  |  |  |  |
| **1** | Identify the study as an economic evaluation and specify the interventions being compared. | Yes | Yes | Yes | Yes | Yes | Yes | Yes | Yes | Yes | Yes | No | Yes |
| **2** | Provide a structured summary that highlights context, key methods, results, and alternative analyses. | Yes | Yes | Yes | Yes | Yes | Yes | Yes | Yes | Yes | Yes | Yes | Yes |
| **3** | Give the context for the study, the study question, and its practical relevance for decision making in policy or practice. | Yes | Yes | Yes | Yes | Yes | Yes | Yes | Yes | Yes | Yes | Yes | Yes |
| **4** | Indicate whether a health economic analysis plan was developed and where available. | No | No | No | No | No | No | No | No | No | No | No | No |
| **5** | Describe characteristics of the study population (such as age range, demographics, socioeconomic, or clinical characteristics). | No | Yes | Yes | Yes | Yes | Yes | Yes | Yes | Yes | No | Yes | Yes |
| **6** | Provide relevant contextual information that may influence findings. | Yes | Yes | Yes | Yes | Yes | Yes | Yes | Yes | Yes | Yes | No | Yes |
| **7** | Describe the interventions or strategies being compared and why chosen. | Yes | Yes | Yes | Yes | Yes | Yes | Yes | Yes | Yes | Yes | Yes | Yes |
| **8** | State the perspective(s) adopted by the study and why chosen. | No | No | Yes | No | Yes | No | Yes | Yes | Yes | Yes | No | Yes |
| **9** | State the time horizon for the study and why appropriate. | Yes | No | Yes | No | Yes | Yes | Yes | Yes | Yes | Yes | Yes | Yes |
| **10** | Report the discount rate(s) and reason chosen. | No | No | Yes | No | Yes | Yes | No | Yes | Yes | Yes | No | Yes |
| **11** | Describe what outcomes were used as the measure(s) of benefit(s) and harm(s). | N/A | N/A | Yes | Yes | N/A | Yes | N/A | Yes | Yes | Yes | N/A | Yes |
| **12** | Describe how outcomes used to capture benefit(s) and harm(s) were measured. | N/A | N/A | Yes | Yes | N/A | Yes | N/A | Yes | Yes | Yes | N/A | Yes |
| **13** | Describe the population and methods used to measure and value outcomes. | N/A | N/A | Yes | Yes | N/A | Yes | N/A | Yes | Yes | Yes | N/A | Yes |
| **14** | Describe how costs were valued. | Yes | Yes | Yes | Yes | Yes | Yes | Yes | Yes | Yes | Yes | Yes | Yes |
| **15** | Report the dates of the estimated resource quantities and unit costs, plus the currency and year of conversion. | Yes | No | Yes | No | Yes | Yes | Yes | Yes | Yes | Yes | Yes | Yes |
| **16** | If modelling is used, describe in detail and why used. Report if the model is publicly available and where it can be accessed. | N/A | N/A | Yes | N/A | Yes | Yes | N/A | Yes | Yes | Yes | N/A | Yes |
| **17** | Describe any methods for analysing or statistically transforming data, any extrapolation methods, and approaches for validating any model used. | No | Yes | Yes | Yes | Yes | Yes | Yes | Yes | Yes | Yes | Yes | Yes |
| **18** | Describe any methods used for estimating how the results of the study vary for subgroups. | No | No | No | Yes | No | Yes | No | No | Yes | No | No | Yes |
| **19** | Describe how impacts are distributed across different individuals or adjustments made to reflect priority populations. | No | No | No | No | No | No | No | No | No | No | No | No |
| **20** | Describe methods to characterise any sources of uncertainty in the analysis. | Yes | No | Yes | Yes | Yes | Yes | No | Yes | Yes | Yes | No | Yes |
| **21** | Describe any approaches to engage patients or service recipients, the general public, communities, or stakeholders (such as clinicians or payers) in the design of the study. | No | No | No | No | No | No | No | No | No | No | No | No |
| **22** | Report all analytic inputs (such as values, ranges, references) including uncertainty or distributional assumptions. | N/A | N/A | Yes | N/A | Yes | Yes | N/A | Yes | Yes | Yes | N/A | Yes |
| **23** | Report the mean values for the main categories of costs and outcomes of interest and summarise them in the most appropriate overall measure. | Yes | Yes | Yes | Yes | Yes | Yes | Yes | Yes | Yes | Yes | Yes | Yes |
| **24** | Describe how uncertainty about analytic judgments, inputs, or projections affect findings. Report the effect of choice of discount rate and time horizon, if applicable. | Yes | No | Yes | Yes | Yes | Yes | Yes | Yes | Yes | Yes | No | Yes |
| **25** | Report on any difference patient/service recipient, general public, community, or stakeholder involvement made to the approach or findings of the study | No | No | No | No | No | No | No | No | No | No | No | No |
| **26** | Report key findings, limitations, ethical or equity considerations not captured, and how these could affect patients, policy, or practice. | Yes | Yes | Yes | Yes | Yes | Yes | Yes | Yes | Yes | Yes | Yes | Yes |
| **27** | Describe how the study was funded and any role of the funder in the identification, design, conduct, and reporting of the analysis | No | No | Yes | Yes | Yes | No | Yes | Yes | Yes | No | No | Yes |
| **28** | Report authors conflicts of interest according to journal or International Committee of Medical Journal Editors requirements. | Yes | Yes | Yes | Yes | Yes | No | Yes | No | Yes | No | Yes | Yes |
| **TOTAL (%)** | | 57 | 48 | 82 | 69 | 80 | 75 | 70 | 79 | 86 | 71 | 48 | 86 |

| **BMJ items** | **Cooper 2022** | **Crean 2024** | **Ferri 2023** | **Hupkens 2020** | **Sawada 2023** |
| --- | --- | --- | --- | --- | --- |
|  |  |  |  |  |  |
| (1) The research question is stated | Yes | Yes | Yes | Yes | Yes |
| (2) The economic importance of the research question is stated | Yes | Yes | Yes | Yes | Yes |
| (3) The viewpoint(s) of the analysis are clearly stated and justified | No | No | No | Yes | No |
| (4) The rationale for choosing the alternative programmes or interventions compared is stated | Yes | Yes | Yes | Yes | Yes |
| (5) The alternatives being compared are clearly described | Yes | Yes | Yes | Yes | Yes |
| (6) The form of economic evaluation used is stated | Yes | No | Yes | Yes | No |
| (7) The choice of form of economic evaluation is justified in relation to the questions addressed | Yes | Yes | Yes | Yes | No |
| (8) The source(s) of effectiveness estimates used are stated | N/A | N/A | Yes | N/A | N/A |
| (9) Details of the design and results of effectiveness study are given (if based on a single study) | N/A | N/A | Yes | N/A | N/A |
| (10) Details of the method of synthesis or meta-analysis of estimates are given (if based on an overview of a number of effectiveness studies) | N/A | N/A | N/A | N/A | N/A |
| (11) The primary outcome measure(s) for the economic evaluation are clearly stated | Yes | No | Yes | Yes | Yes |
| (12) Methods to value health states and other benefits are stated | N/A | N/A | Yes | N/A | N/A |
| (13) Details of the subjects from whom valuations were obtained are given | N/A | N/A | Yes | N/A | N/A |
| (14) Productivity changes (if included) are reported separately | N/A | N/A | N/A | N/A | N/A |
| (15) The relevance of productivity changes to the study question is discussed | N/A | N/A | No | N/A | N/A |
| (16) Quantities of resources are reported separately from their unit costs | Yes | Yes | No | Yes | No |
| (17) Methods for the estimation of quantities and unit costs are described | Yes | Yes | Yes | Yes | Yes |
| (18) Currency and price data are recorded | Yes | Yes | No | Yes | Yes |
| (19) Details of currency of price adjustments for inflation or currency conversions are given | No | No | No | No | No |
| (20) Details of any model used are given | N/A | N/A | N/A | N/A | N/A |
| (21) The choice of model used and the key parameters on which | N/A | N/A | N/A | N/A | N/A |
| (22) Time horizon of costs and benefits is stated | Yes | Yes | No | Yes | Yes |
| (23) The discount rate(s) is stated | No | No | No | No | No |
| (24) The choice of rate(s) is justified | No | No | No | No | No |
| (25) An explanation is given if costs or benefits are not discounted | No | No | No | No | No |
| (26) Details of statistical tests and confidence intervals are given for stochastic data | No | No | Yes | Yes | No |
| (27) The approach to sensitivity analysis is given | N/A | N/A | N/A | N/A | N/A |
| (28) The choice of variables for sensitivity analysis is justified | N/A | N/A | N/A | N/A | N/A |
| (29) The ranges over which the variables are varied are stated | N/A | N/A | N/A | N/A | N/A |
| (30) Relevant alternatives are compared | Yes | Yes | Yes | Yes | Yes |
| (31) Incremental analysis is reported | N/A | N/A | Yes | N/A | N/A |
| (32) Major outcomes are presented in a disaggregated as well as aggregated form | No | Yes | No | Yes | No |
| (33) The answer to the study question is given | Yes | Yes | Yes | Yes | Yes |
| (34) Conclusions follow from the data reported | Yes | Yes | Yes | Yes | Yes |
| (35) Conclusions are accompanied by the appropriate caveats | Yes | Yes | Yes | Yes | Yes |
| **TOTAL (%)** | 68 | 64 | 64 | 82 | 55 |

| **Philips items** | **Cui 2022** | **Gani 2018** | **Hendriks 2016** | **Miller 2020** | **Rao 2017** | **Rodriguez-Pascual 2022** | **Wurschi 2024** |
| --- | --- | --- | --- | --- | --- | --- | --- |
|  |  |  |  |  |  |  |  |
| **S1:** Is there a clear statement of the decision problem/objective problem? | Yes | Yes | Yes | Yes | Yes | Yes | Yes |
| **S1:** Is the objective of the evaluation and model specified and consistent with the stated decision problem? | Yes | Yes | Yes | Yes | Yes | Yes | Yes |
| **S1:** Is the primary decision maker specified? | No | No | No | Yes | No | No | No |
| **S2**: Is the perspective of the model stated clearly? | Yes | Yes | No | Yes | Yes | Yes | Yes |
| **S2**: Are the model inputs consistent with the stated perspective? | Yes | Yes | No | Yes | Yes | Yes | Yes |
| **S2**: Has the scope of the model been stated and justified? | Yes | Yes | Yes | Yes | Yes | Yes | Yes |
| **S2**: Are the outcomes of the model consistent with the perspective, scope, and overall objective of the model? | Yes | Yes | Yes | Yes | Yes | Yes | Yes |
| **S3**: Has the evidence regarding the model structure been described? | Yes | Yes | Yes | Yes | Yes | Yes | Yes |
| **S3:** Is the structure of the model consistent with a coherent theory of the health condition under evaluation | Yes | No | Yes | Yes | Yes | No | Yes |
| **S3**: Have any competing theories regarding model structure been considered? | No | No | No | No | No | No | No |
| **S3**: Are the sources of data used to develop the structure of the model specified? | Yes | Yes | Yes | Yes | Yes | Yes | Yes |
| **S3**: Are the casual relationships described by the model structure justified appropriately? | Yes | Yes | Yes | Yes | Yes | Yes | Yes |
| **S4**: Are the structural assumptions transparent and justified? | Yes | Yes | Yes | Yes | Yes | Yes | No |
| **S4**: Are the structural assumptions reasonable given the overall objective, perspective, and scope of the model? | Yes | Yes | Yes | Yes | Yes | Yes | Yes |
| **S5**: Is there a clear definition of the options under evaluation? | Yes | Yes | Yes | Yes | Yes | Yes | Yes |
| **S5**: Have all feasible and practical options been evaluated? | Yes | Yes | Yes | Yes | Yes | Yes | Yes |
| **S5**: Is there justification for the exclusion of feasible options? | N/A | N/A | N/A | N/A | N/A | N/A | N/A |
| **S6:** Is the chosen model type appropriate given the decision problem and specified casual relationships within the model? | Yes | Yes | Yes | Yes | Yes | Yes | Yes |
| **S7:** Is the time horizon of the model sufficient to reflect all important differences between options? | Yes | Yes | Yes | Yes | Yes | Yes | Yes |
| **S7**: Is the time horizon of the model, and the duration of treatment and treatment effect described and justified? | Yes | Yes | Yes | Yes | Yes | Yes | Yes |
| **S7**: Has a lifetime horizon been used? If not, has a shorter time horizon been justified? | Yes | Yes | Yes | Yes | Yes | Yes | No |
| **S8**: Do the disease states (state transition model) or the pathways (decision tree model) reflect the underlying biological process of the disease in question and the impact of interventions? | Yes | Yes | Yes | Yes | Yes | No | Yes |
| **S9**: Is the cycle length defined and justified in terms of the natural history of the disease? | Yes | N/A | Yes | Yes | No | No | No |
| **D1:** Are the data identification methods transparent and appropriate given the objectives of the model? | Yes | Yes | Yes | Yes | Yes | Yes | Yes |
| **D1**: Where choices have been made between data sources, are these justified appropriately? | No | No | No | Yes | Yes | No | Yes |
| **D1:** Has particular attention been paid to identifying data for the important parameters in the model? | Yes | Yes | Yes | Yes | Yes | Yes | Yes |
| **D1**: Has the process of selecting key parameters been justified and systematic methods used to identify the most appropriate data? | No | No | No | Yes | Yes | No | Yes |
| **D1:** Has the quality of the data been assessed appropriately? | No | No | No | No | No | No | No |
| **D1**: Where expert opinion has been used, are the methods described and justified? | N/A | N/A | Yes | N/A | N/A | N/A | N/A |
| **D2:** Are the pre-model data analysis methodology based on justifiable statistical and epidemiological techniques? | Yes | No | No | Yes | Yes | No | Yes |
| **D2a:** Is the choice of baseline data described and justified? | Yes | No | Yes | Yes | Yes | Yes | Yes |
| **D2a**: Are transition probabilities calculated appropriately? | Yes | Yes | Yes | Yes | Yes | Yes | Yes |
| **D2a**: Has a half cycle correction been applied to both cost and outcome? | Yes | No | No | No | No | No | No |
| **D2a**: If not, has this omission been justified? | N/A | No | No | No | No | No | No |
| **D2b:** If relative treatment effects have been derived from trial data, have they been synthesised using appropriate techniques? | Yes | Yes | Yes | Yes | Yes | No | Yes |
| **D2b:** Have the methods and assumptions used to extrapolate short-term results to final outcomes been documented and justified? Have alternative assumptions been explored through sensitivity analysis? | Yes | No | Yes | Yes | Yes | No | N/A |
| **D2b:** Have assumptions regarding the continuing effect of treatment once treatment is complete been documented and justified? Have alternative assumptions been explored through sensitivity analysis? | N/A | N/A | Yes | Yes | Yes | Yes | Yes |
| **D2c**: Are the utilities incorporated into the model appropriate? | Yes | N/A | Yes | Yes | Yes | Yes | Yes |
| **D2c:** Is the source for the utility weights referenced? | Yes | N/A | Yes | Yes | Yes | Yes | Yes |
| **D2c**: Are the methods of derivation for the utility weights justified? | Yes | N/A | Yes | Yes | Yes | No | Yes |
| **D3:** Have all data incorporated into the model been described and referenced in sufficient detail? | Yes | Yes | Yes | Yes | Yes | Yes | Yes |
| **D3**: Has the use of mutually inconsistent data been justified (i.e. are assumptions and choices appropriate)? | N/A | Yes | N/A | N/A | Yes | No | N/A |
| **D3**: Is the process of data incorporation transparent? | Yes | Yes | Yes | Yes | Yes | Yes | Yes |
| **D3**: If data have been incorporated as distributions, has the choice of distribution for each parameter been described and justified? | Yes | N/A | N/A | Yes | Yes | Yes | N/A |
| **D3**: If data have been incorporated as distributions, is it clear that second order uncertainty is reflected? | Yes | N/A | N/A | Yes | Yes | Yes | N/A |
| **D4**: Have the four principal types of uncertainty been addressed? | No | No | No | No | Yes | No | No |
| **D4**: If not, has the omission of particular forms of uncertainty been justified? | No | No | No | No | N/A | No | No |
| **D4a**: Have methodological uncertainties been addressed by running alternative versions of the model with different methodological assumptions? | Yes | No | Yes | No | Yes | No | No |
| **D4b**: Is there evidence that structural uncertainties have been addressed via sensitivity analysis? | Yes | Yes | Yes | Yes | Yes | Yes | No |
| **D4c**: Has heterogeneity been dealt with by running the model separately for different sub-groups? | No | No | No | No | Yes | No | Yes |
| **D4d**: Are the methods of assessment of parameter uncertainty appropriate? | Yes | Yes | Yes | Yes | Yes | Yes | Yes |
| **D4d**: Has probabilistic sensitivity analysis been done. If not, has this been justified? | Yes | No | Yes | Yes | Yes | Yes | No |
| **D4d:** If data are incorporated as point estimates, are the ranges used for sensitivity analysis stated clearly and justified? | Yes | No | Yes | Yes | Yes | Yes | No |
| **C1:** Is there evidence that the mathematical logic of the model has been tested thoroughly before use? | No | No | No | No | No | No | No |
| **C2**: Are the conclusions valid given the data presented? | Yes | Yes | Yes | Yes | Yes | Yes | Yes |
| **C2:** Are any counterintuitive results from the model explained and justified? | N/A | N/A | N/A | N/A | N/A | N/A | N/A |
| **C2:** If the model has been calibrated against independent data, have any differences been explained and justified? | Yes | N/A | Yes | Yes | N/A | N/A | Yes |
| **C2**: Have the results of the model been compared with those of previous models and any differences in results explained? | Yes | No | No | No | N/A | No | No |
| **TOTAL (%)** | 83 | 60 | 72 | 81 | 87 | 61 | 69 |
